# I-CONECT RESULTS: ENHANCING SOCIAL INTERACTIONS IMPROVES COGNITIVE FUNCTION IN SOCIALLY ISOLATED PARTICIPANTS

**DOI:** 10.1093/geroni/igac059.1585

**Published:** 2022-12-20

**Authors:** Hiroko Dodge, Chao-Yi Wu, Kexin Yu, Peter Lichtenberg, Laura Struble, Kathleen Potempa, Lisa Silbert

**Affiliations:** Oregon Health & Science University, Portland, Oregon, United States; Oregon Health & Science University, Portland, Oregon, United States; Oregon Health & Science University, Palo Alto, California, United States; Wayne State University, Detroit, Michigan, United States; University of Michigan, Ann Arbor, Michigan, United States; University of Michigan, Ann Arbor, Michigan, United States; Oregon Health & Science University, Portland, Oregon, United States

## Abstract

Epidemiological studies suggest that social isolation is a risk factor of dementia although the underlying mechanisms are not well known. A core component of social isolation is a lack of conversational interactions. We recently completed a NIH-funded multi-center randomized controlled trial called Internet-Based Conversational Engagement Clinical Trial (I-CONECT, ClinicalTrials.gov: NCT02871921), which aimed to examine whether social engagement, specifically conversational interactions through webcam and internet, could improve cognitive function in socially isolated older adults with mild cognitive impairment (MCI) or normal cognition. The data was un-blinded in August, 2021. We found strong evidence of efficacy in the primary (global cognitive function, Cohen’s d = 0.73, p=0.03) and secondary (memory function, Cohen’s d=0.67, p=0.03) outcomes at Month 6 (high-dose post-trial endpoint) and Month 12 (maintenance-dose post-trial-endpoint), respectively, using the mixed model for repeated measures. We will present the background, COVID-19 pandemic related protocol modifications and the primary results of this intervention project.

